# Identification and characterization of important residues in the catalytic mechanism of CMP-Neu5Ac synthetase from *Neisseria meningitidis*

**DOI:** 10.1111/j.1742-4658.2010.07696.x

**Published:** 2010-07

**Authors:** Louise E Horsfall, Adam Nelson, Alan Berry

**Affiliations:** 1 Astbury Centre for Structural Molecular Biology, University of LeedsUK; 2 School of Chemistry, University of LeedsUK

**Keywords:** CMP-Neu5Ac, enzyme kinetics, *N*-acylneuraminate cytidylyltransferase, sialic acid

## Abstract

Sialylated oligosaccharides, present on mammalian outer-cell surfaces, play vital roles in cellular interactions and some bacteria are able to mimic these structures to evade their host’s immune system. It would be of great benefit to the study of infectious and autoimmune diseases and cancers, to understand the pathway of sialylation in detail to enable the design and production of inhibitors and mimetics. Sialylation occurs in two stages, the first to activate sialic acid and the second to transfer it to the target molecule. The activation step is catalysed by the enzyme CMP-Neu5Ac synthetase (CNS). Here we used crystal structures of CNS and similar enzymes to predict residues of importance in the CNS from *Neisseria meningitidis*. Nine residues were mutated to alanine, and the steady-state enzyme kinetic parameters were measured using a continuous assay to detect one of the products of the reaction, pyrophosphate. Mutations that caused the greatest loss in activity included K142A, D211A, D209A and a series of mutations at residue Q104, highlighted from sequence-alignment studies of related enzymes, demonstrating significant roles for these residues in the catalytic mechanism of CNS. The mutations of D211A and D209A provide strong evidence for a previously proposed metal-binding site in the enzyme, and the results of our mutations at residue Q104 lead us to include this residue in the metal-binding site of an intermediate complex. This suggests that, like the sugar-activating lipopolysaccharide-synthesizing CMP-2-keto-3-deoxy-*manno*-octonic acid synthetase enzyme KdsB, CNS recruits two Mg^2+^ ions during the catalytic cycle.

## Introduction

Eukaryotic cell-surface glycoconjugates often terminate in a sialic acid molecule, a nine-carbon α-keto acid of which *N*-acetylneuraminic acid (Neu5Ac) is the most abundant [1,2]. Regardless of whether the sialylated oligosaccharides are joined to lipids or proteins they play vital roles in cellular interactions. However, because they are used as recognition markers for ‘self’ cells they have also been implicated in tumour growth and in autoimmune diseases, and a number of pathogenic bacteria use these structures to increase their virulence, mimicking their eukaryotic host’s antigens in order to evade the immune response [3].

The sialylation of sugars occurs in two stages. First, a CMP-*N*-acetylneuraminate synthetase (CNS), also known as *N*-acylneuraminate cytidylyltransferase (EC 2.7.7.43), activates the sialic acid by nucleophilic attack of the O2 atom of Neu5Ac onto the α-phosphate of CTP in a Mg^2+^-dependent ordered-sequential mechanism to yield CMP-Neu5Ac [4–7]. Second, a sialyltransferase adds the activated sialic acid molecule to a sugar with control of both the regio- and stereo-specificities of the reaction [8,9].

The only other sugar activated in a similar manner (i.e. by coupling to a monophosphonucleotide rather than to a diphosphonucleotide) is 2-keto-3-deoxy-*manno*-octonic acid (Kdo) in a reaction performed by the CMP-Kdo synthetase (CKS) enzyme [3-deoxy-manno-octulosonate cytidylyltransferase (EC2.7.7.38)] [10,11]. CNS and CKS share only about 20% amino acid sequence identity [12] but both enzymes exhibit a similar α/β-domain fold, with the major deviation being at the interface region, where the active sites are located [12,13]. However, despite these differences, binding of the nucleotide substrate is similar in the two enzymes, as demonstrated by the locations and conformations of nucleotide analogues in the crystal structures of CNS (PDB 1EYR), ‘capsule-specific’ CKS (K-CKS) (PDB 1GQC and 1GQ9) or lipopolysaccharide-synthesizing CKS (L-CKS) (PDB 3K8D) [13,14].

**Fig. 1:**
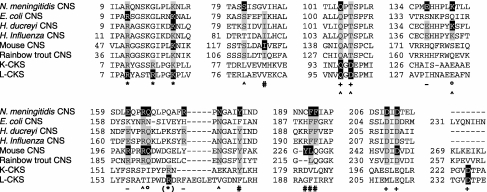
Partial amino acid sequence alignment of CNS enzymes from *Neisseria meningitidis* [23], *Escherichia coli* [29], *Haemophilus ducreyi* [20]*, Haemophilus influenza* [30], mouse [31] and rainbow trout [24], and the two types of CKS enzymes from *E. coli* [32–34]. Sequences were aligned using the ClustalW program [35]. Residues highlighted in black have been identified as important in the literature and have roles assigned [12–18,20]: *, CTP-binding residue of the P-loop; (*), CTP-binding residue; ^, Neu5Ac-binding residue; #, Neu5Ac-binding residue forming part of the hydrophobic pocket; °, residue required in the quaternary organization of the molecule; -, residue lining the active site; +, Mg^2+^-binding residue. Residues highlighted in grey share identity with those highlighted in black.

Previous studies have identified Lys19 in the *Haemophilus ducreyi* CNS, and Lys21 and Arg12 in CNS from *Escherichia coli* (Fig. 1) as important catalytic residues, indicating a role in binding the nucleotide into the active site [15,16]. The crystal structure of CNS from *Neisseria meningitidis* crystallized in the presence of the substrate analogue CDP confirmed the interaction between these residues and the first substrate [12].

**Fig. 2:**
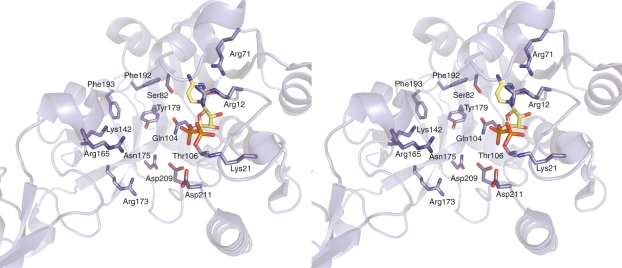
Cross-eye stereo view of the active site of CNS from *Neisseria meningitidis* (blue) with CDP (yellow) in the active site (PDB 1EYR) showing the residues highlighted previously in the literature and those mutated in this study.

Mutation of Arg199, Arg202 or Gln203 to alanine in murine CNS was shown to abolish activity [17]. Similarly, mutation of the equivalent *N. meningitidis* residues (R165A and Q166A; Fig. 1) resulted in enzymes with either no, or strongly reduced, activity [17]. *In silico* docking simulations revealed that Arg165 forms a salt bridge with the carboxylate of the second substrate, Neu5Ac, whereas Gln166 does not interact with either substrate but is required in the quaternary organization of the enzyme [12,17]. This docking also highlighted several other residues (Ser82, Gln104, Thr106, Lys142, Arg165, Tyr179, Phe192 and Phe193) as important in binding the sugar, Neu5Ac [12]. Some of these residues are conserved in the structures of murine CNS and the related CKS enzymes (see Figs 1 and 2) [11,18,19].

**Fig. 3:**
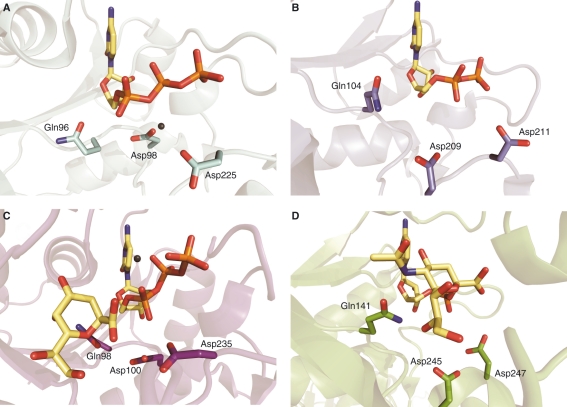
Metal-binding residues of CNS and related enzymes. (A) Gln96, Asp225 and Asp98 in the active site of K-CKS (cyan) (PDB 1GQ9) [13]; (B) Gln104, Asp211 and Asp209 in the *Neisseria meningitidis* enzyme (blue) (PDB 1EYR) [12]; (C) Gln98, Asp235 and Asp100 in L-CKS (purple) (PDB 3K8D) [14] (in addition, a second metal-binding site has been modelled in this enzyme coordinated by the β- and γ-phosphates of the CTP); and (d) the metal-binding residues deduced from the alignment in the structure of the murine CNS (green) (PDB 1QWJ) [18]. When present in the crystal structures, CTP, CDP, Kdo and CMP-Neu5Ac in the active sites are coloured by atom type with the carbon set as yellow. Mg^2+^ is coloured in grey.

Crystal structures of CNS have revealed that the enzyme undergoes significant structural changes on substrate binding: the CNS from *N. meningitidis* was crystallized in an ‘open’ conformation, which allows entry of the second substrate [12], whereas the murine CNS was crystallized in a ‘closed’ conformation with the product CMP-Neu5Ac in the active site [11]. Such movements are expected to be critical in the correct positioning of catalytic residues as well as the divalent metal ions required for catalysis [8]. The *N. meningitidis* CNS is fully active only in the presence of Mg^2+^ ions [5]. Despite this, no electron density has been reported for metal ions in any CNS enzyme structure [12,18]. By contrast, in the X-ray structure of the K-CKS enzyme from *E. coli*, a Mg^2+^ ion and a hydroxide ion were apparent [11,13]. The Mg^2+^ ion was held in place by the bound CTP molecule and residues Asp225 and Asp98. We propose that these residues correspond to Asp209 and Asp211 in the *N. meningitidis* CNS, and Fig. 3 shows these active-site residues in comparison with equivalent residues in the structures of the murine CNS, K-CKS and L-CKS [13,14,18]. Furthermore, we propose that the *N. meningitidis* CNS residue Gln104 corresponds to the L-CKS residue Gln98, which has recently been proposed as a Mg^2+^-binding ligand in a product complex, in addition to its initial role in direct ligation of the sugar OH group in an analogous manner to that found in the DNA/RNA polymerase reaction mechanism [14]. We therefore believe that the mechanism proposed by Heyes *et al*. for the L-CKS enzyme, involving two Mg^2+^ ions in the enzyme active site and the nucleophile O2 being created directly by chelation to the catalytic metal ion [14], can also be applied to CNS enzymes.

In order to probe these possible roles in substrate binding and catalysis, a series of nine *N. meningitidis* CNS alanine-substitution mutants were created at residues Gln104, Lys142, Arg173, Asn175, Tyr179, Phe192, Phe193, Asp209 and Asp211. We chose to concentrate on these residues because they appear to contact the *N*-acetyl group or the glyceryl moiety of Neu5Ac, and we believe these more functionalized areas of the molecule are likely to confer the ability to differentiate Neu5Ac from other sugars, or that they are residues proposed to form the binding site of the catalytic Mg^2+^ ion. We have determined the kinetic parameters of the wild-type enzyme from *N. meningitidis* using a continuous spectrophotometric assay measuring the release of pyrophosphate during the reaction, and by comparing the kinetic parameters determined for the alanine mutants we were able to identify key catalytic residues and put forward a revised catalytic mechanism for CNS.

## Results and Discussion

In order to make comparisons with the constructed mutant CNS enzymes, we first measured the steady-state kinetic parameters of wild-type CNS by following the rate of formation of the product pyrophosphate, using a continuous spectrophotometric assay, as recently used to determine the kinetics of an L-CKS enzyme [14]. Control experiments in the absence of the second substrate (Neu5Ac) showed no significant production of pyrophosphate, confirming that the assay was detecting true activity rather than nonproductive hydrolysis of the CTP. The enzyme showed typical ordered-sequential kinetic behaviour; it did not bind Neu5Ac in the absence of CTP, verified by isothermal titration calorimetry (data not shown), and Lineweaver–Burk plots of initial rate against CTP concentration at various fixed Neu5Ac concentrations, and vice versa, converged. We found some inhibition of the enzyme activity at high concentrations of CTP, but values of the true steady-state parameters –*V*_max_, *K*_m(CTP)_ and *K*_m(Neu5Ac)_– were obtained by fitting the initial rates of reaction, at varying concentrations of both substrates, to the overall rate equation for a Bi-Bi ordered sequential mechanism (Table 1). The *K*_m(CTP)_ of 17 μm is in line with that measured for the *H. ducreyi* CNS [20], but significantly lower than that measured for a number of other bacterial CNS enzymes. However, we note that our assay was a continuous enzyme assay rather than the discontinuous assays often used in the initial characterizations and that, like the *H. ducreyi* measurements, our assays were carried out at physiologically relevant pH (pH 7.5), rather than at higher pH values (pH 8.5-9.0), which were used in the earlier discontinuous assays [5,20–22]. In addition, the earlier assays measured the apparent kinetic values for Neu5Ac at constant CTP concentrations above those at which we saw substrate inhibition of the enzyme, accounting for the other differences observed for the values of *K*_m(Neu5Ac)_ and *k*_cat_ [5,23] (Table 1).

**Table 1:** True kinetic parameters determined for wild-type and mutant CNS. True kinetic parameters, *k*_cat_, *K*_m(CTP)_ and *K*_m(Neu5Ac)_ were obtained by fitting initial rates of reactions measured at varying concentrations of CTP and Neu5Ac to the appropriate ordered-sequential Bi-Bi rate equation using nonlinear regression.

	*k*_cat_ (min^−1^)	*K*_m(CTP)_ (μm)	*K*_m(Neu5Ac)_ (μm)	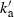 (μm)	*k*_cat_/*K*_m_ (μm^−1^·min^−1^) CTP	*k*_cat_/*K*_m_ (μm^−1^·min^−1^) Neu5Ac
WT	610 ± 4	17 ± 1	68 ± 1	190 ± 5	36	8.9
Q104A	130 ± 2	31 ± 2	2600 ± 90	180 ± 8	4.3	0.051
R173A	420 ± 3	48 ± 1	290 ± 5	320 ± 7	8.6	1.4
N175A	1500 ± 20	270 ± 8	1600 ± 50	550 ± 20	5.4	0.93
Y179A	3.1 ± 0.2	40 ± 10	110 ± 40	290 ± 100	0.077	0.028
F192A	1200 ± 6	150 ± 3	380 ± 3	2400 ± 20	7.9	3.1
F193A	300 ± 5	52 ± 3	780 ± 20	310 ± 10	5.8	0.38
D211A	0.041 ± 0.003	26 ± 5	66 ± 10	37 ± 10	1.6 × 10^−3^	6.2 × 10^−4^
D209A	Insufficient activity to determine the kinetic parameters when 50 nmol of enzyme present					

The necessity of including a sulfhydryl reagent, such as dithiothreitol, in the assay reaction to give the full activity of the enzyme has previously been reported, and such reagents are generally added to assays [5,8,23]. In contrast, we found no such addition necessary. The CNS enzymatic activity, as measured by apparent values of *k*_cat_ and *K*_m(Neu5Ac)_, at a fixed CTP concentration of 154 μm and five different Neu5Ac concentrations, were 560 ± 30 s^−1^ and 190 ± 20 μm, respectively, in the presence of 0.2 mm dithiothreitol, compared with 540 ± 10 s^−1^ and 130 ± 9 μm in its absence.

**Fig. 4:**
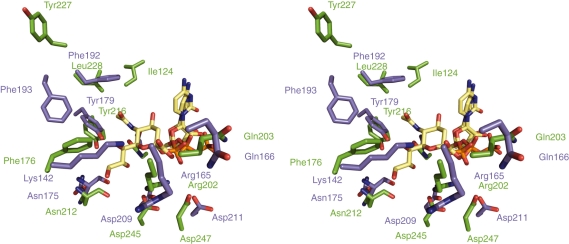
Cross-eye stereo-view of the residues of interest (blue) in the active site of the CNS from *Neisseria meningitidis* containing CDP (yellow) (PDB 1EYR) [12] superimposed with their equivalent residues (green) of the murine CNS containing CMP-Neu5Ac (yellow) (PDB 1QWJ) [18]. All residues are numbered, with the exception of Gln104 in the *N. meningitidis* CNS equivalent to Gln141 in the murine enzyme, which are shown at the back of the view behind the sialic acid molecule.

In order to identify residues in the wild-type *N. meningitidis* CNS with potential roles in substrate discrimination or in forming the binding site for the Mg^2+^ ion required in catalysis, we studied both sequence and structural alignments. Structural alignment (Fig. 4) of the *N. meningitidis* CNS active site containing CDP [12] onto the murine CNS active site containing CMP-Neu5Ac (PDB code 1QWJ) [18] suggested that the substrates for the two enzymes would bind in very similar positions and orientations [12], and we therefore identified nine residues in the CNS crystal structure (PDB 1EYR) – Gln104, Lys142, Arg173, Asn175, Tyr179, Phe192, Phe193, Asp209 and Asp211 – which we believed to be important in binding Neu5Ac. Alanine replacement mutations were created separately at each of these positions, the enzymes were purified and the effect of mutation was determined kinetically (Tables 1 and 2).

### Enzyme to *N*-acetyl contacts

Tyr179, Phe192 and Phe193 are proposed to interact with the methyl group of the *N*-acetyl moiety of Neu5Ac, forming a hydrophobic pocket that allows the methyl group of the *N*-acetyl moiety to bind into what is otherwise a very polar active site [12]. Mutations of Phe192 and Phe193 to alanine were constructed and the kinetic parameters of the purified F192A and F193A mutant enzymes were obtained in the same way as the wild-type enzyme (Table 1). The results support the role of these residues in binding Neu5Ac, because the mutants have *k*_cat_ values comparable to that of the wild-type enzyme but *K*_m_ values indicative of a ‘poorer’ substrate with lower affinity for the enzyme active site. The mutation of Tyr179 (Y179A) also significantly affects the kinetic parameters for the enzyme, although, in this case, the major alterations appear as a 200-fold decrease in *k*_cat_ rather than substantial changes in *K*_m_ for either substrate (Table 1). This result might be caused by a mis-positioning of the substrate in the active site, or to other consequences of the enzyme mechanism (see below). Mosimann *et al.* [12] also propose that the hydrophobic pocket plays a role in substrate discrimination against binding of the 5-OH group of Kdo and in favour of the *N*-acetyl group of Neu5Ac and, although these residues are conserved in CNS enzymes, none is conserved in the related CKS enzymes. In addition, we found that 2-keto-3-deoxy-d-glycero-d-galacto-nonulosonic acid (Kdn), a sialic acid that only differs from Neu5Ac by possessing a hydroxyl group at position 5, rather than an *N*-acetyl, has a *k*_cat_*/K*_m_ value around 5000 times lower than the natural substrate, Neu5Ac. This contrasts with the murine enzyme, which has a less pronounced pocket consisting of Ile124, Leu228 and Tyr227, and which exhibits only a 15-fold lower activity [18,24]. More significantly, the only sialic acid-activating enzyme reported to exhibit a preference for Kdn over Neu5Ac is an enzyme from rainbow trout, which has yet to be structurally resolved. The sequence alignment in Fig. 1 suggests that the rainbow trout enzyme has both the Ile113 and Leu216 residues of the murine hydrophobic pocket, while the Tyr is absent [24,25]. Therefore, evidence clearly suggests the requirement for three hydrophobic residues in forming a binding pocket that allows the enzyme a preference for Neu5Ac over Kdn.

### Enzyme to glyceryl moiety contacts

In the docking studies of Mosimann *et al.* [12], the hydroxyl group of Tyr179 lies within hydrogen-bonding distance of O7 and O9 of the glyceryl moiety of Neu5Ac, and a network of noncovalent bonds between enzyme and substrate is proposed in the active site upon substrate binding [12]. This network is important in correctly positioning the substrate, active-site metal ions and the associated water or hydroxide ions [6] ready for catalysis. The kinetic parameters determined for the Y179A mutant (Table 1), showing a 200-fold decrease in its *k*_cat_ value, demonstrate that the hydroxyl group of Tyr179 plays an important role in catalysis, supporting the view that an organized hydrogen-bonding network around this residue may be important.

The same region of the Neu5Ac, namely the O9 atom, lies at the base of the binding pocket formed by the backbone atoms of Asn175 [12,18] (Fig. 4). Although site-directed mutations cannot change the nature of the backbone, we nevertheless mutated Asn175 to alanine to see the effect of the mutation. The N175A mutation had surprising effects, with increases in the *K*_m_ values for both CTP (16-fold) and Neu5Ac (23-fold) (Table 1). It seems most likely that the packing of the side-chain of this residue holds the backbone in an orientation that stabilizes the active conformation of the whole active site and that a change in this packing can be felt throughout the active site.

When the residue Arg173 was mutated to alanine, a small increase, of less than five-fold, was seen in the *K*_m_ of the enzyme (Table 1). The small difference is a result of the residue being relatively far from both substrates whilst still making up part of the active site (Fig. 4). By contrast, the mutation of Lys142 to alanine produces an enzyme with extremely low levels of activity. Experiments with as much as 0.33 mg of enzyme per assay, varying CTP concentration in the presence of a fixed concentration of Neu5Ac of 0.615 mm, allowed us to estimate a value for the apparent *K*_m_ for CTP and the apparent *k*_cat_ for the reaction. These values were compared with values for the wild-type enzyme measured under identical conditions (Table 2). This revealed a 10 000-fold reduction in *k*_cat_ with an insignificant, fourfold, rise in *K*_m_ for CTP. Despite running enzyme assays with up to 41 mm Neu5Ac in the presence of a fixed concentration of CTP of 0.154 mm, it was not possible to saturate the enzyme, and only an estimate of the *k*_cat_/*K*_m_ for Neu5Ac could be made from the gradient of the line obtained in the initial rate versus the Neu5Ac concentration plot. This showed a value 100 000-fold lower than for the wild-type enzyme.

**Table 2:** Apparent kinetic parameters determined for wild-type and mutant CNS. Apparent kinetic parameters, *k*_cat_, *K*_m(CTP)_ and *K*_m(Neu5Ac)_ were obtained by fitting initial rates of reactions measured at varying concentrations of one substrate, at a fixed concentration of the other, to the Michaelis–Menten equation using nonlinear regression. The fixed concentrations were [CTP] = 0.154 mm and [Neu5Ac] = 0.615 mm.

	*k*_cat(app)_ (min^−1^) CTP	*K*_m(app)_ (μm) CTP	*k*_cat(app)_/*K*_m(app)_ (μm^−1^·min^−1^) CTP	*k*_cat(app)_ (min^−1^) Neu5Ac	*K*_m(app)_ (μm) Neu5Ac	*k*_cat(app)_/*K*_m(app)_ (μm^−1^·min^−1^) Neu5Ac
WT	540 ± 30	34 ± 5	16	540 ± 10	130 ± 9	4.1
Q104A	–	–	–	110 ± 9	4700 ± 900	0.024
Q104L	0.17 ± 0.01	150 ± 20	1.1 × 10^−3^	–	–	2.9 × 10^−4^ ± 1 × 10^−5^
Q104E	0.046 ± 0.001	200 ± 9	2.3 × 10^−4^	0.027 ± 0.001	310 ± 40	8.7 × 10^−5^
Q104N	0.74 ± 0.03	32 ± 4	0.023	–	–	7.5 × 10^−4^ ± 2 × 10^−5^
K142A	0.056 ± 0.002	140 ± 10	3.8 × 10^−4^	–	–	3.6 × 10^−5^ ± 2 × 10^−6^

**Fig. 5:**
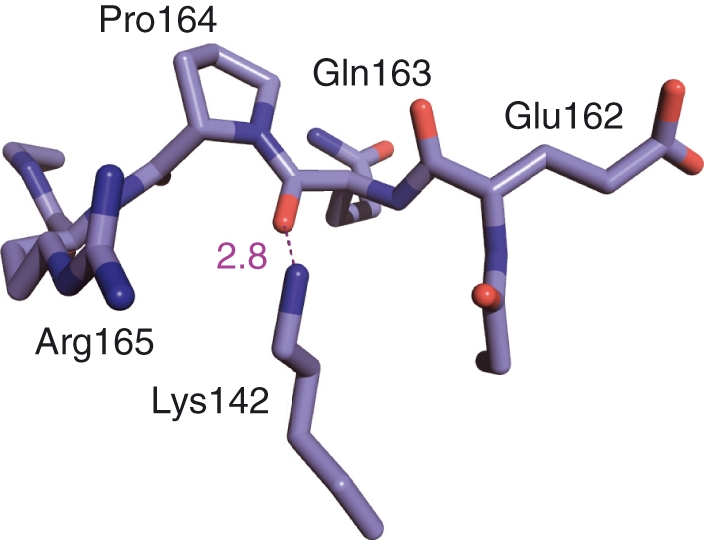
The interactions of residue Lys142 in the CNS from *Neisseria meningitidis* (blue). The hydrogen bond formed with the backbone of Gln163 is shown in pink with the distance given in angstroms.

CNS is a dimeric enzyme, with the enzyme active site being composed of residues from both subunits. Lys142 from one subunit contributes to the active site of the other subunit, and we therefore investigated whether its mutation (K142A) had affected either the folding or dimerization of the polypeptide chain. The CD spectrum of the mutant was identical to that of the wild-type enzyme, and gel filtration showed that, like the wild-type enzyme, the mutant enzyme was present as a dimer, thus confirming that the K142A mutant had folded and dimerized correctly (data not shown). Lys142 is semiconserved in the CNS family, also being found in the enzymes from *Haemophilus influenzae* and *H. ducreyi* [20]. Lys142 has been proposed to interact with the O7 and/or the O9 of the glyceryl part of Neu5Ac [12] but its role is not clear, partly because of the lack of detail on Neu5Ac binding in CNS enzymes. Our findings suggest a major role in catalysis for Lys142. The small changes in Michaelis constants for substrates in the K142A mutant, coupled with the distance estimated between the modelled position of Neu5Ac and Lys142, suggests to us an indirect role in obtaining the correct active-site geometry for activity. We propose that Lys142 is vital in positioning residue Arg165 so that it forms a salt bridge with the carboxylate of the Neu5Ac substrate [12]. Munster *et al.* [17] have previously shown that the R165A mutation creates an enzyme with no activity and that mutation of the neighbouring Gln166 to alanine also reduces the activity strongly. The crystal structure of CNS in complex with CDP [12] shows that this section of the polypeptide is intimately involved in the active site – Glu162 is part of the enzyme active site [12] and the neighbouring Gln163 plays a role in controlling the position of residue 165 because the residue in position 164 is proline. Gln163 is, in turn, positioned by its backbone hydrogen bonding to Lys142 (Fig. 5). We believe that the mutation K142A therefore not only limits the possibility of its own interactions with the substrate, but, more importantly, prevents Arg165 from being correctly positioned to neutralize the negative charge of the carboxylate group of the second substrate, sialic acid, resulting in the incorrect alignment of substrates for catalysis and hence the significant decreases in *k*_cat_ observed.

### Metal-binding-site mutations

Previous work has proposed that the metal-binding site of the *N. meningitidis* CNS is composed of residues Asp209 and Asp211 [12]. The X-ray crystal structure of the K-CKS from *E. coli*, which carries out the same reaction on a similar substrate, has provided further evidence for the location of the Mg^2+^ ion in the CNS enzyme [13]. This structure reveals that the metal-binding site is composed of residues Asp98 and Asp225, placed similarly to residues Asp209 and Asp211 in the *N. meningitidis* CNS, despite the lack of agreement in the sequence alignment of the CNS and CKS enzymes for these residues (Fig. 1) [13]. Recently, in the enzyme L-CKS, Gln98 (equivalent to Gln104 in the *N. meningitidis* enzyme) has been proposed as a third metal-binding residue [14], binding a single Mg^2+^ ion in the product complex. The structural similarity between these residues in CNS, K-CKS and L-CKS is shown in Fig. 3. We investigated the role of these three residues using site-directed mutagenesis.

**Fig. 6:**
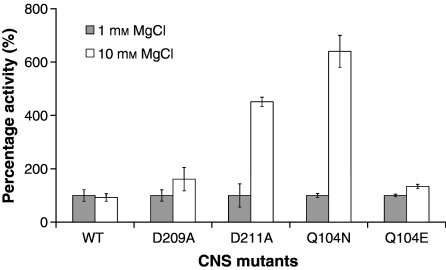
The effect of increased [Mg^2+^] on enzyme activity. Percentage change of activity of wild-type CNS and mutants upon the addition of MgCl_2_ to a concentration of 10 mm. The rate of reaction for each variant using 154 μm CTP and 615 μm Neu5Ac in standard reaction buffer (1 mm MgCl_2_) was taken as 100%.

The D209A mutation showed the greatest effect, eliminating the enzymatic activity to such a degree that the kinetic parameters could not be determined (Table 2). Similarly, the D211A mutation had a crippling effect on activity. In this case we were able to measure the kinetic parameters for the mutant enzyme (Table 2). While these showed minor effects on the *K*_m_ for either substrate, the *k*_cat_ for the reaction was decreased 15 000-fold. These findings strongly support a major role for Asp209 and Asp211 in the coordination of the catalytically critical Mg^2+^ ion. The two aspartic acid residues would interact with the β- and γ-phosphate groups of CTP, as is common in nucleotide-binding enzymes [12]. Magnesium has been shown to be an essential cofactor in the enzyme CNS [5] and is typically included in our assays at a concentration of 1 mm. Because mutation of metal-binding residues would be expected to reduce the affinity of the enzyme for Mg^2+^, we decided to investigate the effect of increasing the Mg^2+^ concentration to 10 mm. Despite previous reports suggesting that 10 mm Mg^2+^ is required for optimal activity [5], this addition had no effect on the level of activity of the wild-type enzyme (Fig. 6). This is probably because of the lower pH of our reaction and because the CTP concentrations used here were much lower than previously used. However, similar treatment of the D211A mutant increased the activity by more than four-fold. Increasing the concentration of Mg^2+^ had little effect on the D209A mutant. These differences perhaps reflect the nature of the binding from the two residues. Asp209 is proposed to make a bidentate interaction with Mg^2+^, whereas Asp211 would make only a monodentate interaction with Mg^2+^ and would also bind the hydroxyl/water molecule ligated to the Mg^2+^ ion, as suggested for its similar residue in the CKS enzymes [13,14].

The role of Gln104 in Mg^2+^ binding and/or catalysis is less clear. In contrast to the major decrease in *k*_cat_ of the enzyme when Asp209 or Asp211 were mutated to alanine, the Q104A mutant manifested its most significant change in the kinetic parameter of the *K*_m_ for Neu5Ac, with only ∼ fivefold and ∼ twofold decreases in *k*_cat_ and *K*_m(CTP)_, respectively, while the *K*_m(Neu5Ac)_ rose by ∼40-fold (Table 1). These findings appear to reflect a significant role in substrate recognition rather than catalysis. Mosimann *et al.* [12] have proposed that this highly conserved residue would interact with the ribose of CTP, and the O8 and N5 positions of Neu5Ac, and that it might be a key residue in discriminating between sialic acid substrates with different functional groups at C6. The small changes in *K*_m_ for CTP on mutation to alanine do not suggest a major role in CTP binding, if one assumes that the *K*_m(CTP)_ is a reflection of the binding affinity for that substrate. However, the significant increase in *K*_m(Neu5Ac)_ would support a role in binding that substrate. In order to investigate this further we made a series of mutations at Gln104. We maintained the length of the side chain at this position while introducing a negative charge by a Q104E mutation, shortened the chain while maintaining H-bonding capacity in a Q104N mutation and provided a hydrophobic residue larger than alanine in a Q104L mutant. All of these proteins were over-expressed successfully and attempts were made to measure the true *k*_cat_ and *K*_m_ values. This proved impossible because of difficulties in saturating the enzyme with Neu5Ac and we resorted to measuring the apparent values of the kinetic parameters at fixed concentrations of the other substrate (Table 2; see above).

**Fig. 7:**
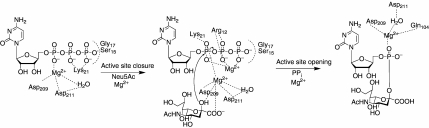
Proposed mechanism of CNS, highlighting the residues involved in accordance with that proposed recently for the L-CKS enzyme from *Escherichia coli*, KdsB [14], using the sequence alignment in Fig. 1 and the structure of the CNS from *Neisseria meningitidis* (PDB 1EYR) [12].

Maintaining the nature of the residue at position 104 (Q104N mutant) resulted in no change to the apparent *K*_m_ for CTP, while the introduction of a charged residue (Q104E) or a larger hydrophobic residue (Q104L) increased the apparent *K*_m_ for CTP by only four- to sixfold. By contrast, these mutations had major effects on the *K*_m_ for Neu5Ac. An accurate estimate for this parameter could only be found for the Q104E mutation because it was not possible to carry out assays with sufficiently high concentrations of Neu5Ac to saturate the other enzymes, and plots of the initial rate versus Neu5Ac concentration were always linear. The Q104E mutation caused the apparent *K*_m_ for Neu5Ac to increase by twofold, but the other mutations caused at least a 100-fold increase. Maintaining the length and hydrogen-bonding potential of the residue at position 104 (Q104E) therefore appears to be critical for Neu5Ac binding, while the introduction of hydrophobic residues (Q104A or Q104L) or shortening the side chain (Q104N) at this position is detrimental. However, we noted that mutations at Gln104 also had significant effects on the *k*_cat_ of the enzyme reaction. While the true *k*_cat_ measured for the Q104A mutant (Table 1) fell only by five-fold, the apparent values measured for the Q104L, Q104E and Q104N mutants fell by between 800- and 20 000-fold, depending on the mutation introduced (Table 2). Together with the changes in *K*_m_ described above, this results in mutant enzymes with *k*_cat_/*K*_m_ values between 200- and 47 000-fold lower than the wild-type enzyme, suggesting a role for Gln104 in catalysis as well as substrate binding. In the related L-CKS enzyme from *E. coli*, KdsB, the equivalent residue to Gln104 is Gln98, which forms a double hydrogen-bond with the sugar ligand [14] but is also postulated to be involved in Mg^2+^ binding after cleavage of the α-β phosphate bond and product formation, when the enzyme adopts a more open, intermediate conformation concomitant with re-orientation of Gln98. Two of the Q104 mutants were also assayed at increased [Mg^2+^], in an effort to ascertain if this altered the mutants’ activity, as shown to be the case for D209A and D211A. The activity of Q104N increased (by more than sixfold) at 10 mm Mg^2+^, whereas the mutant Q104E was much less affected by the metal addition. We believe that our results support a dual role for a residue in this position (Gln104), as proposed by Heyes *et al.* [14] in the related enzyme, L-CKS, because mutation would interfere with both Neu5Ac binding as well as playing a role in the re-orientation of Mg^2+^ binding during the re-opening of the active site after catalysis allowing product release (Fig. 7).

## Conclusions

Using the continuous spectrophotometric assay we have determined accurate kinetic data for the wild-type CNS from *N. meningitidis*. Mutagenic studies have allowed further insight into the roles played by certain residues in the catalysis of the reaction to produce CMP-Neu5Ac. CNS requires a hydrophobic pocket to aid binding of Neu5Ac via the methyl of the *N*-acetyl group; Tyr179 to form crucial interactions with the glyceryl chain of Neu5Ac; Lys142 to position essential residues via a hydrogen-bonding network and metal-binding residues; and requires Asp211 and Asp209 to bind the catalytic Mg^2+^ ion. Our results also lend weight to a recently suggested mechanism for the L-CKS enzymes involving two metal ions in the enzyme’s active site [14]. In accordance with this, we propose (Fig. 7) a mechanism for CNS enzymes related to that of L-CKS with two active-site metal ions. In this mechanism, both Mg^2+^ ions would play a role in correctly orientating the substrates and activating the α-phosphate of CTP, whereas the catalytic Mg^2+^ ion activates the sugar hydroxyl group. In this mechanism we propose that this ion does not remain in a fixed position, as previously presumed [6,7], but has an altered ligation position upon the enzyme adopting a more open conformation after cleavage of the α-β phosphate bond, which allows product release. The increased mechanistic understanding gained from this study should allow incremental advances in the design and production of inhibitors and mimetics of CNS and other enzymes in the pathways to complex carbohydrates.

## Materials and methods

### Materials

All chemicals were obtained from Sigma Aldrich (Dorset, UK) unless stated otherwise, with the exception of Neu5Ac and CTP which were from Carbosynth (Compton, UK), yeast extract, tryptone, ampicillin, isopropyl thio-β-d-galactoside and dithiothreitol which were from Melford Laboratories (Ipswich, UK), and chelating Sepharose™ fast-flow resin which was from GE Healthcare (Little Chalfont, Bucks, UK). Kdn was synthesized in accordance with published protocols [26,27].

### Mutant creation

The gene encoding the CMP-NeuAc synthetase from *N. meningitidis*, with a (His)_6_ tag at the C-terminus, in the plasmid pCW(ori+) was a kind gift from Michel Gilbert (Ontario, Canada) [23]. Mutations were introduced using a QuikChange® Lightning Site-Directed Mutagenesis Kit supplied by Agilent Technologies (South Queensferry, UK), using primers designed as directed.

### Enzyme purification

The wild-type and mutant enzymes were over-expressed in Electro10 blue or XL10 Gold cells (Agilent Technologies) and grown at 37 °C in Luria–Bertani (LB) medium containing 50 mg·L^−1^ of ampicillin and 0.1 mm isopropyl thio-β-d-galactoside. Cells were harvested after 16 h by centrifugation and were lysed, using a cell disruptor from Constant Systems Ltd. (Daventry, UK), in buffer containing 20 mm Tris/HCl (pH 7.5), 0.5 m NaCl and 20 mm imidazole. Cell debris was removed by centrifugation at 30 000 ***g*** using a Beckman Coulter Avanti J-26 XP centrifuge (Beckman Coulter, High Wycombe, UK). The enzymes were purified from the crude lysate by addition to nickel-charged resin, successive washes with buffer containing 20 mm Tris/HCl (pH 7.5), 0.5 m NaCl and 20 mm imidazole, and elution into buffer containing 20 mm Tris/HCl (pH 7.5), 0.5 m NaCl and 500 mm imidazole. All protein samples were purified to homogeneity, as judged by SDS/PAGE, and dialysed into 20 mm Tris/HCl (pH 7.4). Protein concentrations were measured using the Bio-Rad protein assay kit II (BioRad Laboratories Ltd, Hemel Hempstead, Herts, UK).

### Enzyme kinetics

The kinetic parameters were determined using the EnzChek® pyrophosphate assay kit (Invitrogen, Paisley, UK) and a Jasco V-560 UV/Vis spectrophotometer (Jasco, Great Dunmow, Essex, UK) to follow the formation of pyrophosphate. The assay uses inorganic pyrophosphatase to convert the pyrophosphate to two molecules of phosphate which are then reacted with 2-amino-6-mercapto-7-methylpurine ribonucleoside enzymatically by purine nucleoside phosphorylase with an accompanying shift in absorbance maximum to 360 nm [28]. The amount of pyrophosphate produced in the reaction was calculated by comparison with a pyrophosphate standard curve. The initial rates of reaction were measured over a range of substrate concentrations in 50 mm Tris/HCl (pH 7.5) containing 1 mm MgCl_2_ and 0.1 mm sodium azide at 22 °C. These data were fitted to Eqn (1) by nonlinear regression using the enzfitter programme (Biosoft, Great Shelford, UK) to obtain values of the true kinetic parameters. 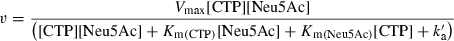
(1) where *v* is the initial rate, *V*_max_ is the maximal rate of the reaction when both substrates are saturating, *K*_m(CTP)_ and *K*_m(Neu5Ac)_ are the true Michaelis constants for CTP and Neu5Ac, respectively, and 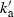 is a constant.

On occasion, when the *K*_m_ of either substrate was too high to be determined by these means the *K*_m(app)_ and *k*_cat(app)_ were found by varying the concentration of one substrate whilst holding the other constant. Values of 154 μm CTP and 615 μm Neu5Ac were used as these constant concentrations, and these initial data were fitted to Eqn (2). 
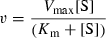
(2) where *v* is the initial rate, *V*_max_ is the maximal rate of the reaction at saturating substrate concentration and *K*_m_ is the apparent *K*_m_ for the variable substrate.

### Effect of dithiothreitol addition

The activity of CNS at a fixed CTP concentration (154 μm) was measured at five different Neu5Ac concentrations in the presence or absence of 0.2 mm dithiothreitol.

### Effect of Mg^2+^ concentration

The effect of an increase in the concentration of Mg^2+^ was determined by measuring the rate of the reaction in triplicate at 154 μm CTP and 615 μm Neu5Ac in the reaction buffer described before and in the reaction buffer containing a 10-fold higher concentration of MgCl_2_ (10 mm). The percentage activity was calculated by setting each mutants’ activity as 100% when in the standard reaction buffer (1 mm MgCl_2_), so the effect of Mg^2+^ addition to all mutants could be represented on a single bar chart.

### Isothermal titration calorimetry (ITC)

ITC experiments were performed using a MicroCal VP-ITC unit (GE Healthcare) at 25 °C. CNS was prepared by dialysis into 200 mm Tris/HCl (pH 9.0) containing 10 mm MgCl_2_, followed by degassing under reduced pressure. The enzyme was present at a concentration of 112.5 μm, Neu5Ac was at 10 mm and CTP was at 10 mm (made up with dialysate). ITC experiments comprised an initial ligand injection of 2 μL followed by 30 injections of 8 μL with a 240 s interval between each titration. The ITC cell volume was 1.41 mL. The initial data point was deleted from the integrated data to allow for equilibration of ligand/receptor at the needle tip. Heats of dilution for the ligands were determined in control experiments, and these were subtracted from the integrated data before curve fitting. Data were fit in Origin 5.0 (MicroCal) with the standard One Site model.

### CD

Data were collected on a Jasco J715 CD spectropolarimeter (Jasco). The CD signal for measurements in the far-UV region (200–260 nm) was recorded in a 1-mm path-length cell with a protein concentration of 0.2 mg·mL^−1^. Far-UV CD spectra were recorded using a 1-nm bandwidth, 1-nm resolution, 20-nm·min^−1^ scan speed and a response time of 8 s, averaging five scans.

### Gel filtration

Two-hundred and fifty microlitres of the wild-type enzyme and 250 μL of the K142A mutant were injected onto a BioSep S-2000 SEC (Phenomenex, Macclesfield, Cheshire, UK) column attached to a BioCAD Vision workstation (Applied Biosystems, Warrington, Cheshire, UK). The samples were run at 1 mL·min^−1^ and the absorbance (A) of the eluate was monitored at 214 nm.

## Abbreviations

CKS: CMP-Kdo synthetase
CNS: CMP-*N*-acetylneuraminate synthetase
K-CKS: capsule-specific CMP-Kdo synthetase
Kdn: 2-keto-3-deoxy-d-glycero-d-galacto-nonulosonic acid
Kdo: 2-keto-3-deoxy-*manno*-octonic acid
L-CKS: lipopolysaccharide-synthesizing CMP-Kdo synthetase
Neu5Ac: *N*-acetylneuraminic acid

## References

[1] Varki A (2007). Glycan-based interactions involving vertebrate sialic-acid-recognizing proteins. Nature.

[2] Schauer R (2000). Achievements and challenges of sialic acid research. Glycoconj J.

[3] Schauer R (2009). Sialic acids as regulators of molecular and cellular interactions. Curr Opin Struct Biol.

[4] Qasba PK, Ramakrishnan B, Boeggeman E (2005). Substrate-induced conformational changes in glycosyltransferases. Trends Biochem Sci.

[5] Warren L, Blacklow RS (1962). The biosynthesis of cytidine 5′-monophospho-*N*-acetylneuraminic acid by an enzyme from *Neisseria meningitidis*. J Biol Chem.

[6] Ambrose MG, Freese SJ, Reinhold MS, Warner TG, Vann WF (1992). ^13^C NMR investigation of the anomeric specificity of CMP-*N*-acetylneuraminic acid synthetase from *Escherichia coli*. Biochemistry.

[7] Samuels NM, Gibson BW, Miller SM (1999). Investigation of the kinetic mechanism of cytidine 5′-monophosphate *N*-acetylneuraminic acid synthetase from *Haemophilus ducreyi* with new insights on rate-limiting steps from product inhibition analysis. Biochemistry.

[8] Mizanur RM, Pohl NL (2008). Bacterial CMP-sialic acid synthetases: production, properties, and applications. Appl Microbiol Biotechnol.

[9] Kean EL (1991). Sialic acid activation. Glycobiology.

[10] Ghalambor MA, Heath EC (1966). The biosynthesis of cell wall lipopolysaccharide in *Escherichia coli.* IV. Purification and properties of cytidine monophosphate 3-deoxy-D-manno-octulosonate synthetase. J Biol Chem.

[11] Jelakovic S, Schulz GE (2001). The structure of CMP:2-keto-3-deoxy-manno-octonic acid synthetase and of its complexes with substrates and substrate analogs. J Mol Biol.

[12] Mosimann SC, Gilbert M, Dombroswki D, To R, Wakarchuk W, Strynadka NC (2001). Structure of a sialic acid-activating synthetase, CMP-acylneuraminate synthetase in the presence and absence of CDP. J Biol Chem.

[13] Jelakovic S, Schulz GE (2002). Catalytic mechanism of CMP:2-keto-3-deoxy-manno-octonic acid synthetase as derived from complexes with reaction educt and product. Biochemistry.

[14] Heyes DJ, Levy C, Lafite P, Roberts IS, Goldrick M, Stachulski AV, Rossington SB, Stanford D, Rigby SE, Scrutton NS (2009). Structure-based mechanism of CMP-2-keto-3-deoxymanno-octulonic acid synthetase: convergent evolution of a sugar-activating enzyme with DNA/RNA polymerases. J Biol Chem.

[15] Tullius MV, Vann WF, Gibson BW (1999). Covalent modification of Lys19 in the CTP binding site of cytidine 5′-monophosphate *N*-acetylneuraminic acid synthetase. Protein Sci.

[16] Stoughton DM, Zapata G, Picone R, Vann WF (1999). Identification of Arg-12 in the active site of *Escherichia coli* K1 CMP-sialic acid synthetase. Biochem J.

[17] Munster AK, Weinhold B, Gotza B, Muhlenhoff M, Frosch M, Gerardy-Schahn R (2002). Nuclear localization signal of murine CMP-Neu5Ac synthetase includes residues required for both nuclear targeting and enzymatic activity. J Biol Chem.

[18] Krapp S, Munster-Kuhnel AK, Kaiser JT, Huber R, Tiralongo J, Gerardy-Schahn R, Jacob U (2003). The crystal structure of murine CMP-5-*N*-acetylneuraminic acid synthetase. J Mol Biol.

[19] Jelakovic S, Jann K, Schulz GE (1996). The three-dimensional structure of capsule-specific CMP: 2-keto-3-deoxy-manno-octonic acid synthetase from *Escherichia coli*. FEBS Lett.

[20] Tullius MV, Munson RS, Wang J, Gibson BW (1996). Purification, cloning, and expression of a cytidine 5′-monophosphate *N*-acetylneuraminic acid synthetase from *Haemophilus ducreyi*. J Biol Chem.

[21] Vann WF, Silver RP, Abeijon C, Chang K, Aaronson W, Sutton A, Finn CW, Lindner W, Kotsatos M (1987). Purification, properties, and genetic location of *Escherichia coli* cytidine 5′-monophosphate *N*-acetylneuraminic acid synthetase. J Biol Chem.

[22] Haft RF, Wessels MR (1994). Characterization of CMP-*N*-acetylneuraminic acid synthetase of group B *streptococci*. J Bacteriol.

[23] Gilbert M, Watson D, Wakarchuk W (1997). Purification and characterization of the recombinant CMP-sialic acid synthetase from *Neisseria meningitidis*. Biotechnol Lett.

[24] Nakata D, Munster AK, Gerardy-Schahn R, Aoki N, Matsuda T, Kitajima K (2001). Molecular cloning of a unique CMP-sialic acid synthetase that effectively utilizes both deaminoneuraminic acid (Kdn) and *N*-acetylneuraminic acid (Neu5Ac) as substrates. Glycobiology.

[25] Terada T, Kitazume S, Kitajima K, Inoue S, Ito F, Troy FA, Inoue Y (1993). Synthesis of CMP-deaminoneuraminic acid (CMP-KDN) using the CTP:CMP-3-deoxynonulosonate cytidylyltransferase from rainbow trout testis. Identification and characterization of a CMP-Kdn synthetase. J Biol Chem.

[26] Fitz W, Schwark JR, Wong CH (1995). Aldotetroses and C(3)-modified aldohexoses as substrates for *N*-acetylneuraminic acid aldolase – A model for the explanation of the normal and the inversed stereoselectivity. J Org Chem.

[27] Watts AG, Withers SG (2004). The synthesis of some mechanistic probes for sialic acid processing enzymes and the labeling of a sialidase from *Trypanosoma rangeli*. Can J Chem.

[28] Webb MR (1992). A continuous spectrophotometric assay for inorganic phosphate and for measuring phosphate release kinetics in biological systems. Proc Natl Acad Sci U S A.

[29] Zapata G, Vann WF, Aaronson W, Lewis MS, Moos M (1989). Sequence of the cloned *Escherichia coli* K1 CMP-*N*-acetylneuraminic acid synthetase gene. J Biol Chem.

[30] Fleischmann RD, Adams MD, White O, Clayton RA, Kirkness EF, Kerlavage AR, Bult CJ, Tomb JF, Dougherty BA, Merrick JM (1995). Whole-genome random sequencing and assembly of *Haemophilus influenzae* Rd. Science.

[31] Munster AK, Eckhardt M, Potvin B, Muhlenhoff M, Stanley P, Gerardy-Schahn R (1998). Mammalian cytidine 5′-monophosphate *N*-acetylneuraminic acid synthetase: a nuclear protein with evolutionarily conserved structural motifs. Proc Natl Acad Sci U S A.

[32] Pazzani C, Rosenow C, Boulnois GJ, Bronner D, Jann K, Roberts IS (1993). Molecular analysis of region 1 of the *Escherichia coli* K5 antigen gene cluster: a region encoding proteins involved in cell surface expression of capsular polysaccharide. J Bacteriol.

[33] Rosenow C, Roberts IS, Jann K (1995). Isolation from recombinant *Escherichia coli* and characterization of CMP-Kdo synthetase, involved in the expression of the capsular K5 polysaccharide (K-CKS). FEMS Microbiol Lett.

[34] Goldman RC, Bolling TJ, Kohlbrenner WE, Kim Y, Fox JL (1986). Primary structure of CTP:CMP-3-deoxy-D-manno-octulosonate cytidylyltransferase (CMP-Kdo synthetase) from *Escherichia coli*. J Biol Chem.

[35] Chenna R, Sugawara H, Koike T, Lopez R, Gibson TJ, Higgins DG, Thompson JD (2003). Multiple sequence alignment with the Clustal series of programs. Nucleic Acids Res.

